# Behavioral features in child and adolescent huntingtin gene‐mutation carriers

**DOI:** 10.1002/brb3.2630

**Published:** 2022-05-23

**Authors:** Erin E. Reasoner, Ellen van der Plas, Hend M. Al‐Kaylani, Douglas R. Langbehn, Amy L. Conrad, Jordan L. Schultz, Eric A. Epping, Vincent A. Magnotta, Peggy C. Nopoulos

**Affiliations:** ^1^ Department of Psychiatry University of Iowa Hospital and Clinics Iowa City Iowa USA; ^2^ Stead Family Children's Hospital at the University of Iowa Iowa City Iowa USA; ^3^ Department of Radiology University of Iowa Hospital and Clinics Iowa City Iowa USA; ^4^ Department of Neurology University of Iowa Hospital and Clinics Iowa City Iowa USA

**Keywords:** anxiety, depression, Huntington disease, neostriatum, neuropsychological tests, pediatric psychology

## Abstract

**Introduction:**

We compared neuropsychiatric symptoms between child and adolescent huntingtin gene‐mutation carriers and noncarriers. Given previous evidence of atypical striatal development in carriers, we also assessed the relationship between neuropsychiatric traits and striatal development.

**Methods:**

Participants between 6 and 18 years old were recruited from families affected by Huntington's disease and tested for the huntingtin gene expansion. Neuropsychiatric traits were assessed using the Pediatric Behavior Scale and the Behavior Rating Inventory of Executive Function. Striatal volumes were extracted from 3T neuro‐anatomical images. Multivariable linear regression models were conducted to evaluate the impact of group (i.e., gene nonexpanded [GNE] or gene expanded [GE]), age, and trajectory of striatal growth on neuropsychiatric symptoms.

**Results:**

There were no group differences in any behavioral measure with the exception of depression/anxiety score, which was higher in the GNE group compared to the GE group (estimate = 4.58, *t*(129) = 2.52, FDR = 0.051). The growth trajectory of striatal volume predicted depression scores (estimate = 0.429, 95% CI 0.15:0.71, *p* = .0029), where a negative slope of striatal volume over time was associated with lower depression/anxiety.

**Conclusions:**

The current findings show that GE children may have lower depression/anxiety compared to their peers. Previously, we observed a unique pattern of early striatal hypertrophy and continued decrement in volume over time among GE children and adolescents. In contrast, GNE individuals largely show striatal volume growth. These findings suggest that the lower scores of depression and anxiety seen in GE children and adolescents may be associated with differential growth of the striatum.

## Graphical abstract

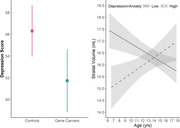

## 1 INTRODUCTION

Huntington's disease (HD) is a fatal, neurodegenerative disorder caused by a CAG repeat expansion in the huntingtin (*HTT*) gene on chromosome 4 (OMIM 143100). Cognitive and psychiatric changes are prominent throughout the course of HD and often manifest prior to onset of motor symptoms (Epping et al., 2016; Paoli et al., 2017; You et al., 2014). Adult carriers can present with executive dysfunction, including difficulties with attention, working memory, planning, and self‐monitoring (Julio et al., 2019; Larsen et al., 2015). Psychiatric symptoms, including depression, anxiety, irritability, apathy, perservations, and obsessions, are also prevalent in pre‐HD adults (Duff et al., 2007; Epping et al., 2016; Martinez‐Horta et al., 2016). However, analyzing young adult carriers over 20 years from estimated motor onset yielded no evidence of neuropsychiatric symptoms (Scahill et al., 2020). Thus, it remains unclear whether these symptoms are present prior to the onset of neurodegeneration. Crucially, *HTT* is active from conception (Barnat et al., 2020; Godin et al., 2010), underscoring the need to evaluate neuropsychiatric symptoms in child and adolescent *mHTT* gene‐mutation carriers in the context of brain development.

The Kids‐HD study was designed to evaluate the impact of *mHTT* on development of brain structure and function in children and adolescents (6–18 years old) who have a family history of HD (van der Plas et al., 2020). Results from the Kids‐HD study demonstrated that *mHTT* affects multiple facets of development, including striatal volume (van der Plas et al., 2019), striatal‐cerebellar circuitry (Tereshchenko et al., 2020), body mass index (Tereshchenko et al., 2020), and cognitive function. The striatum is of primary pathological importance in HD (Aylward et al., 2011), and we demonstrated that *mHTT* carriers (gene‐expanded [GE]; CAG repeat >36) exhibited atypical striatal development compared to their peers who did not inherit the mutation (gene nonexpanded [GNE]; CAG repeat ≤36). Rather than following the neurotypical pattern of overall volume increase in childhood (with subsequent minor volume loss in adolescence), the GE group demonstrated striatal hypertrophy before the age of 10 followed by continued volume loss throughout the observed age range—a negative slope of change in striatal volume. These results point to the importance of evaluating trajectories rather than static phenotypes, where the former focuses on pattern of change and the latter evaluates phenotypes at a single timepoint. Prior studies have demonstrated trajectories of brain development to be a sensitive predictor of neuropathology (Giedd & Rapoport, 2010).

Variation in the number of CAG repeats can affect phenotypic expression, referred to as ‘‘dose effects’’ (Schultz, Saft, et al., 2021). Longer repeat lengths are typically associated with higher disease burden (van der Plas et al., 2019). However, prior to disease onset, having a CAG repeat in the low pathogenic range (approximately up to 43 repeats) may correspond with better cognitive performance, compared to CAG repeats in the nonpathological range (Schultz, Saft, et al., 2021; Schultz, van der Plas, et al., 2021). The impact of CAG repeat on neuropsychiatric symptoms in child and adolescent carriers has yet to be evaluated.

Neuropsychiatric traits are strong predictors of quality of life in HD (Banaszkiewicz et al., 2012; Brugger et al., 2015; Fritz et al., 2018), and it is crucial to establish a better understanding of their development. The present analysis evaluated differences in parent‐rated neuropsychiatric symptoms between GE and GNE. We also investigated the impact of CAG repeat length on these symptoms. Lastly, we evaluated the associations between neuropsychiatric symptoms and age‐related developmental trajectory of the striatum.

## 2 METHODS

### 2.1 Participants

**FIGURE 1:**
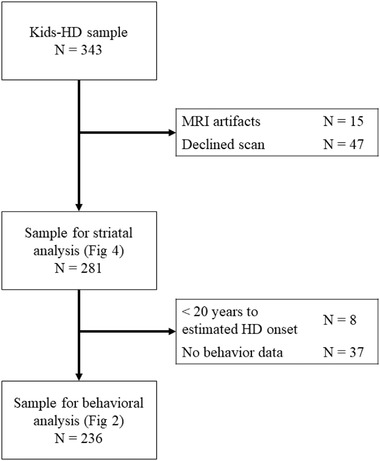
Consort Diagram. Of the initial Kids‐HD sample, 281 observations included usable magnetic resonance imaging (MRI) data (82%), composing the sample of our previous analysis (van der Plas et al., 2019). Two‐hundred thirty‐six of these observations were over 20 years from onset and included behavioral data for subsequent analysis (an 84% overlap between samples)

The Kids‐HD study enrolled children and adolescents between 6 and 18 years of age who had a biological parent and/or grandparent with HD (van der Plas et al., 2019). Eligible participants had to be asymptomatic at the time of recruitment. Individuals with a history of neurological disorders, head trauma, or brain surgery were excluded from the study. The Kids‐HD study uses an accelerated longitudinal design where participants were recruited between the ages of 6–18 years old, and some returned for repeated visits while others were assessed once. Data were collected between May 2009 and January 2018. The current analysis overlaps with previously published data (van der Plas et al., 2019), with the exception that we excluded participants here who did not have complete neurocognitive data (Figure 1). Additionally, to maximally capture developmental processes rather than early phases of degeneration, we limited our sample to individuals with over 20 years to predict disease onset postparticipation. Years to disease onset was estimated for GE individuals based on CAG repeat length and age at time of data collection using a previously developed model (Langbehn et al., 2004).

### 2.2 Genetic analysis

Blood or saliva samples were obtained from all participants to determine GE or GNE status. PCR analysis, conducted at the University of Iowa Molecular Diagnostic Laboratory, was used to detect CAG expansion size in exon 1 of both HTT alleles (van der Plas et al., 2019). As part of the Kids‐HD pipeline design (van der Plas et al., 2020), samples were collected for research purposes only and were not entered into medical records. All participants, family members, researchers, and clinical staff who had direct contact with participants remained blind to the participant's gene status. Rather, genetic testing results were de‐identified and accessed only by research staff who had no contact with participants. Furthermore, participants were required to have an age‐appropriate understanding of their familial risk for HD.

### 2.3 Parent‐rated measures of behavior

Neuropsychiatric traits were assessed using two parent‐reported questionnaires: the Pediatric Behavior Scale (PBS) Short Form and the Behavior Rating Inventory of Executive Function (BRIEF). These measures provide an indication of the child's ‘‘traits’’ rather than their ‘‘state’’: the PBS specifies these behaviors as a characteristic, rather than a specific episode, and the BRIEF asks about behaviors during the past 6 months.

The PBS Short Form is a 30‐item version of the original 165‐item assessment and consists of four domains: Opposition/Aggression, Hyperactivity/Inattention, Depression/Anxiety, and Physical Health. Questions are rated on a four‐point Likert scale ranging from 0 (“almost never or not at all”) to 3 (“very often or very much”) (Lindgren & Koeppl, 1987). The PBS‐30 was normalized using a sample of 600 children between the ages of 6–12 and has previously been used to evaluate behavioral traits of children in various medical populations (Brumbaugh et al., 2016; McCarthy et al., 2003; Nopoulos et al., 2010).

The BRIEF is an 86‐question parent‐reported assessment that measures eight clinical scales of executive function in children and adolescents (Roth et al., 2014). The Global Executive Composite is divided into the indices of Behavioral Regulation (defined as impulse control, flexibility, and emotional control) and Metacognition (defined as organizational and planning, self‐monitoring, and problem‐solving abilities, as well as working memory). Responses are rated on a three‐point Likert scale ranging from 0 (“never”) to 2 (“often”).

For both measures, raw scores were converted to standardized T‐scores (mean = 50; SD = 10), where higher scores represent greater impairment.

### 2.4 Socioeconomic status

Family socioeconomic status (SES) was quantified using a modified Hollingshead scale. Income categories were categorized into ordinal scores, with lower values indicating lower SES (Hollingshead, 1975).

### 2.5 MRI acquisition and processing

Prior to June 2016, participants (N = 180 visits) completed neuroimaging with a 3T Siemens Trio TIM (Siemens AG, Munich, Germany), while after June 2016 participants (N = 56 visits) were scanned on a 3T General Electric Discovery MR750w (GE Medical Systems, Chicago, IL). Sixty‐two additional observations were excluded from all analyses due to missing MRI data, either because the participant declined the scan (N = 47) or because of MRI motion artifacts (N = 15). Anatomical T1‐weighted images were acquired with 1.1 mm isotropic resolution, as described previously (van der Plas et al., 2019). Realtime prospective motion correction (PROMO) was employed to reduce movement related artifacts (White et al., 2010).

Details regarding image processing were also published previously (van der Plas et al., 2019). Briefly, Advanced Normalization Tools (ANTs) were used to correct for intensity inhomogeneity, and images were subsequently processed using the BrainsTools software, which is a robust method to address multiscanner‐induced variation in regional anatomical volume (Young Kim & Johnson, 2013). Brain regions were labeled using the joint label fusion approach (Wang & Yushkevich, 2013), where labels based on the Desikan–Killiany–Tourville atlas were mapped to the subject native space. The striatum was the region of interest (ROI) that was selected a priori. Volumes from both hemispheres were combined.

### 2.6 Statistical analysis

Demographics of the sample were summarized with descriptive statistics, including chi‐square tests and Fisher's exact tests.

To compare neuropsychiatric symptoms between GE and GNE participants, separate models were run for each PBS/BRIEF domain. Age and sex were included as co‐variates in all models. The sex × group interaction was evaluated in each model, but only included if it was statistically significant. Note that random effects for participants and family members were added to account for nonindependency of observations.

To evaluate the impact of CAG on behavioral outcomes, we conducted multivariable mixed linear regressions with behavioral scores as the dependent variables, and CAG repeat, age and sex as predictors. Models were run separately for GNE versus GE, and random effects for participants and family were included.

Finally, we determined associations between neuropsychiatric symptoms and striatum trajectory, with the latter being expressed as an age × striatum interaction. Other predictors included group (GNE vs. GE), age and sex, as well as random effects for participants and family. Again, age × sex interaction was included in the model only if it was significant. Analyses conducted under this aim were limited to neuropsychiatric symptoms for which a statistically significant group difference was observed.

The false discovery rate (FDR) was calculated to correct for repeated analysis, and models with FDR <0.10 were considered significant (Benjamini & Hochberg, 1995). All analyses were performed in R version 4.1.0.

### 2.7 Standard protocol approvals, registrations, and patient consents

All procedures and communications conducted as part of this study followed the written protocol approved by the University of Iowa Hospitals and Clinics Institutional Review Board (ClinicalTrials.gov Identifier: NCT01860339). Consent and/or assent was obtained from all participants, and informed consent was obtained from a parent or guardian. All measures were obtained for research purposes only, and participants consented to nondisclosure of the results (van der Plas et al., 2019).

## 3 RESULTS

### 3.1 Sample demographics

Fifty‐nine GE individuals and 91 GNE individuals, who provided 91 and 145 observations, respectively, were included in the final sample (Table 1). Across the entire sample, 46 had two visits (30.67%), and 23 had three or more visits (15.33%), with no differences in the distribution of visits across groups (*t*(130) = 0.05, *p* = .96).

**TABLE 1:** Demographics across groups (number of observations)

Variables and summary statistiscs	GNE (N = 145)	GE (N = 91)
Age (years)		
Mean (SD)	12.7 (3.28)	12.8 (3.40)
Median [minimum, Maximum]	13.3 [6.08, 17.8]	13.2 [6.00, 17.8]
Sex		
Female	76.0 (52.4%)	58.0 (63.7%)
Male	69.0 (47.6%)	33.0 (36.3%)
CAG repeat length		
Mean (SD)	20.3 (4.02)	43.4 (3.42)
Median [minimum, Maximum]	19.0 [15.0, 34.0]	43.0 [36.0, 51.0]
Socioeconomic status		
1	0 (0%)	1.00 (1.1%)
2	67.0 (46.2%)	43.0 (47.3%)
3	64.0 (44.1%)	36.0 (39.6%)
4	12.0 (8.3%)	10.0 (11.0%)
5	2.00 (1.4%)	1.00 (1.1%)

*Note*: GNE, gene‐non‐expanded (participants with a family history of Huntington's disease who did not inherit the mutant allele); GE, Gene‐Expanded (carriers of the mutant allele).

Mean age at evaluation was 12.72 (SD = 3.3), and groups did not differ in mean age at evaluation (*t*(186) = −0.39, *p* = .70). On average, GE participants were estimated to be 37.28 years from motor onset, with predicted years to onset ranging from 20 to 90 years. The distribution of sex and SES were also similar between groups, (*p* = .11; *p* = .71, respectively).

Twenty‐five participants (10.5%) were on at least one psychotropic medication at the time of the study. There was no difference between groups in the number of individuals who reported using medication for attention‐deficit disorder (*χ* ^2^ (1, N = 236) = 0, *p *= 1.00) or anti‐depressants/anti‐anxiolytics (*χ* ^2^ (1, N = 236) = 2.17, *p* = .14).

### 3.2 Behavioral outcomes in GE and GNE

Table 2 shows the sex and age effects for all seven behavioral measures of the BRIEF and PBS. Older age was associated with lower scores on the following domains (i.e., negative age estimate): BRIEF Global Executive Composite (estimate = −0.58, *t*(231) = −2.32, FDR = 0.032) and Behavior Regulation Index (estimate = −0.055, *t*(230) = −2.38, FDR = 0.032) as well as PBS Physical Health (estimate = 0.41, *t*(195) = 1.81, FDR = 0.096), Hyperactivity/Inattention (estimate = −0.78, *t*(205) = −3.16, FDR = 0.007), and Aggression/Opposition (estimate = −0.44, *t*(232) = −2.07, FDR = 0.080).

**TABLE 2:** Effects of age and sex on Pediatric Behavior Scale (PBS) and Behavior Rating Inventory of Executive Function (BRIEF) measures

	Age					Sex				
Outcome measure	*β*	SE	*t‐*Value (*df*)	*p*‐Value	FDR	*β*	SE	*t*‐Value (df)	*p*‐Value	FDR
Global Executive Composite	−0.58	0.25	*t*(231) = −2.32	.021	0.032	4.24	1.56	*t*(226) = 2.72	.007	0.020
Behavior Regulation	−0.55	0.23	*t*(230) = −2.38	.018	0.032	1.80	1.43	*t*(214) = 1.27	.207	0.207
Metacognition	−0.25	0.26	*t*(216) = −0.95	.343	0.343	4.17	1.68	*t*(232) = 2.48	.014	0.020
Physical Health	0.41	0.23	*t*(195) = 1.81	.072	0.096	0.40	1.47	*t*(224) = 0.27	.784	0.784
Depression/ Anxiety	−0.01	0.23	*t*(220) = −0.05	.962	0.962	−2.77	1.49	*t*(232) = −1.86	.255	0.255
Hyperactivity/ Inattention	−0.78	0.25	*t*(205) = −3.16	.002	0.007	2.36	1.58	*t*(229) = 1.49	.274	0.274
Aggression/ Opposition	−0.44	0.21	*t*(232) = −2.07	.084	0.080	0.93	1.32	*t*(222) = 0.70	.644	0.644

*Note*: Sex × group effect was excluded from subsequent models.

Abbreviations: FDR, false discovery rate; SE, standard error.

For both BRIEF Global Executive Composite and Metacognition, males scored higher than did females (estimate = 4.24, *t*(226) = 2.72, FDR = 0.020; estimate = 4.17, *t*(232) = 2.48, FDR *= *0.020, respectively). Sex was not associated with any PBS domains. The sex × group interaction was dropped from the analyses as it was not associated with BRIEF or PBS scores (all *p *> .10).

**FIGURE 2:**
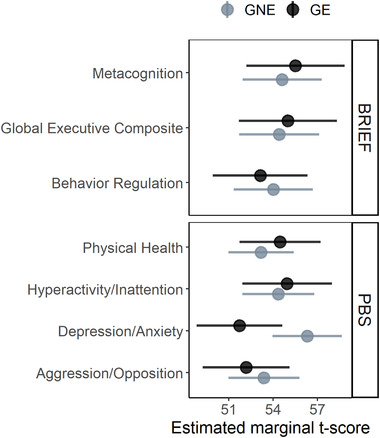
Parent‐behavioral measures in gene‐expanded (GE) and gene nonexpanded (GNE) groups. Mean age‐ and sex‐adjusted estimates for Behavior Rating Inventory of Executive Function (BRIEF) and Pediatric Behavior Scale‐30 (PBS) *t*‐scores in GNE (light gray) and GE (dark gray) groups were determined via linear mixed effects model, while controlling for the random effect*s* of family relations and repeated visits. Filled circles indicate the marginal means and the underlying lines indicate the 95% confidence interval

Table 3 shows the group comparison for all seven behavioral measures of the BRIEF and PBS. There were no significant group differences, with the exception of the PBS Depression/Anxiety score where the mean score for GNE participants was 4.58 points higher than in GE participants (*t*(129) = 2.52, FDR = 0.051; Figure 2).

**TABLE 3:** Effects of group on Pediatric Behavior Scale (PBS) and Behavior Rating Inventory of Executive Function (BRIEF) measures

	GNE		GE		Summary statistics			
Outcome measure	EMM	95% CI	EMM	95% CI	Diff means	*t*‐Value (*df*)	*p*‐Value	FDR
Global Executive Composite	54.66	51.96:57.37	55.25	51.90:58.61	−0.587	*t*(132) = −0.292	.770	0.770
Behavior Regulation	54.15	51.47:56.82	53.27	50.03:56.51	0.880	*t*(116) = 0.474	.636	0.770
Metacognition	54.83	52.16:57.50	55.73	52.36:59.10	−0.905	*t*(132) = −0.433	.665	0.770
Physical Health	53.28	51.07:55.49	54.57	51.77:57.36	−1.290	*t*(124) = −0.738	.462	0.671
Depression/ Anxiety	56.17	53.82:58.52	51.59	48.63:54.54	4.580	*t*(129) = 2.520	.013	0.051
Hyperactivity/ Inattention	54.49	52.05:56.92	55.09	52.00:58.17	−0.598	*t*(122) = −0.308	.759	0.759
Aggression/ Opposition	53.47	51.07:55.87	52.27	49.29:55.24	1.200	*t*(139) = 0.671	.503	0.671

*Note*: All models accounted for age and sex effects.

Abbreviations: 95% CI, 95%, confidence interval; Diff means, difference in group means; EMM, estimated marginal means; GE, gene‐expanded group; GNE, gene nonexpanded group.

Post hoc analyses were conducted to further explore the observed difference in Depression/Anxiety between groups. Since PBS scores were on a T‐score metric (mean = 50, SD = 10), we can make comparisons to a normative sample (Lindgren & Koeppl, 1987). For the current analyses, impairment was defined as having a score of T ≥70, which corresponds to scores that were ≥ 98th percentile. As such, 2% of the normative sample is expected to fall within this score range (Lindgren & Koeppl, 1987). By comparison, within the GNE group, 11.7% had impaired scores, which was higher than the expected percentage of the normative sample (*t*(144) = 7.17, *p* = 3.69 × 10^−11^). Of note, the proportion of GE individuals with impaired scores (4.40%) was not different from the normative sample (*t*(90) = −0.28, *p =* .78).

### 3.3 Impact of CAG repeat

No significant associations were observed between CAG repeat and any of the outcome measures in the GNE or GE group (Table 4).

**TABLE 4:** Impact of CAG repeat length on Pediatric Behavior Scale (PBS) and Behavior Rating Inventory of Executive Function (BRIEF) measures

	GNE					GE				
Outcome Measure	*ß*	SE	*t‐*Value (*df*)	*p‐*Value	FDR	*ß*	SE	*t*‐Value (*df*)	*p*‐Value	FDR
Global Executive Composite	−0.15	0.33	*t*(87.1) = −0.45	.652	0.652	−0.65	0.46	*t*(57.7) = −1.42	.161	0.602
Behavior Regulation	−0.19	0.32	*t*(99.7) = −0.60	.553	0.652	−0.21	0.43	*t*(60.5) = −0.49	.629	0.652
Metacognition	−0.18	0.32	*t*(71.8) = −0.58	.564	0.652	−0.61	0.48	*t*(54.4) = −1.30	.201	0.603
Physical Health	−0.01	0.25	*t*(55.4) = −0.03	.979	0.979	−0.44	0.42	*t*(54.1) = −1.04	.302	0.702
Depression/ Anxiety	−0.28	0.30	*t*(74.6) = −0.94	.351	0.702	−0.12	0.37	*t*(59) = −0.31	.759	0.979
Hyperactivity/ Inattention	−0.46	0.30	*t*(86.2) = −1.56	.123	0.702	0.07	0.47	*t*(57.7) = 0.14	.887	0.979
Aggression/ Opposition	−0.34	0.30	*t*(101) = −1.18	.241	0.702	−0.21	0.41	*t*(58.0) = −0.51	.612	0.979

*Note*: All models accounted for age and sex effects. *p*‐Value adjusted for false discovery rate.

Abbreviations: FDR, false discovery rate; GE, gene‐expanded group; GNE, gene nonexpanded group; SE, standard error.

### 3.4 Age‐related change of striatum and depression/anxiety

**FIGURE 3:**
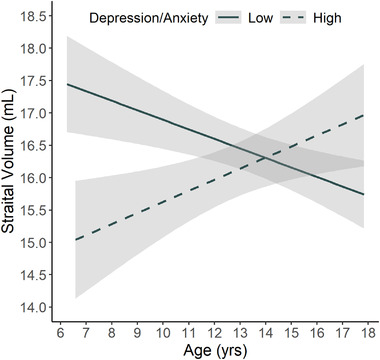
Age‐dependent effect of striatal volume on Anxiety/Depression trait. Smoothed age‐dependent change of striatal volume in low‐Depression/Anxiety (solid) and high‐Depression/Anxiety (dashed) groups. Combined observations from all gene expanded (GE) and gene nonexpanded (GNE) were used to create a distribution of Pediatric Behavior Scale‐30 (PBS) Anxiety/Depression *t*‐scores. For visualization purposes only, this plot shows striatal trajectory from the lower (≤45; *n* = 70) and upper quantiles (≥60.25; *n* = 59) of the sample distribution, independent of gene status

Regarding the relationship of depression and anxiety scores with striatal development, there was an age × striatal volume interaction (estimate = 0.43, 95% CI 0.15:0.71, *p *= .0029), where striatal growth with increasing age (i.e., a positive slope) was associated with higher depression scores, while a reduction of the striatum with increasing age (i.e., negative slope) was associated with lower depression scores, independent of group. For illustration purposes, this association was visualized using extreme depression/anxiety groups composed of both GE and GNE individuals. Individuals with low anxiety/depression (T scores within the lowest 25% of the distribution) exhibited a *reduction* in striatal volume as they aged (Figure 3). By contrast, individuals with high anxiety/depression (T scores in the top 25% of the distribution) exhibited *growth* of striatal volume as they aged (Figure 3).

**FIGURE 4:**
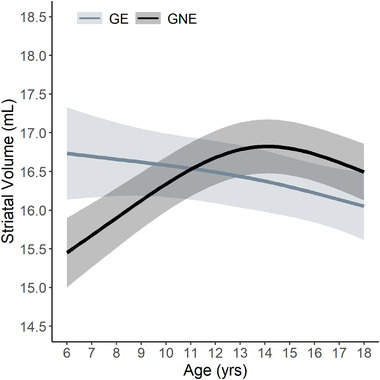
Age‐dependent change in striatal volume by group (adapted from van der Plas et al., 2019). Previous analysis of the Kids‐HD dataset revealed a significant group difference in age‐dependent change of striatal volume. The patterns of overall striatal growth among gene nonexpanded individuals (GNE; N = 162; light gray) and loss of striatal volume among gene‐expanded individuals (GE; N = 119; dark gray) reflect the differential trajectories of high and low depression/anxiety groups observed in the present analysis

The contrast between high and low anxiety/depression groups recapitulates our previous work evaluating the difference of striatal growth trajectory between the GE and GNE groups, which is shown in Figure 4 (van der Plas et al., 2019). Between ages 6 and 18, GE participants have a negative slope of change where there is early striatal hypertrophy followed by continual decline in volume. In contrast, the GNE growth trajectory shows a mostly positive slope of change in volume with increases early in the age range and a slight decline after adolescence (representing the normal maturational process of pruning (Sowell et al., 2002).

## 4 DISCUSSION

The current study evaluated behavioral manifestations of executive function and psychiatric symptoms in child and adolescent *mHTT* gene‐mutation carriers and noncarriers. While most measures showed no significant group differences, we found that the GE group had significantly lower depression/anxiety scores, suggesting a protective effect of mHTT. Moreover, the trajectory of striatal development was associated with depression/anxiety scores, where individuals with a negative trajectory of striatal volume (as seen in GE participants) had lower depression/anxiety scores compared to those who have a positive slope of striatal volume (as seen in GNE participants). The pattern of growth and development of the striatum is the defining feature differentiating GNE and GE groups (van der Plas et al., 2019), and the findings here suggest that this pattern may be associated with reduced risk of anxiety/depression in child and adolescent carriers.

Post hoc analysis of depression and anxiety symptoms in the GE group revealed a similar neuropsychiatric burden to what was reported in the PBS normative sample (Lindgren & Koeppl, 1987). This is in agreement with prior studies where both young adult carriers greater than 20 years to estimated onset, and adult carriers over 10 years to onset reportedly show no significant differences in depression or anxiety compared to healthy controls without a family history of HD (Scahill et al., 2020; Tabriz et al., 2009). Rather, depression symptoms and suicidal ideation appear to peak shortly after motor manifestation of Huntington's disease (Craufurd et al., 2001; Epping & Paulsen, 2011; Epping et al., 2013). Onset of symptoms is often attributed to the psychological burden of receiving predictive testing results and subsequent onset of motor symptoms (Licklederer et al., 2008). Since individuals included in this analysis were all children and adolescences estimated to be over 20 years from HD onset, our findings clarify the evolution of anxiety/depression symptoms in HD and support the notion of neuropsychiatric onset prior to the prodromal phase.

Given established psychosocial effects of HD, it is pertinent to consider the possible influence of environmental factors on the observed group difference. All participants were recruited from families that struggle with the emotional, financial, and practical impact of HD. Still, we also found that our GE individuals were significantly more likely to have a known gene positive parent, while most GNE individuals had only an affected grandparent. To account for this potential environmental difference, we repeated our analysis with only those participants who had a gene positive parent. Within this subsample, the GNE group continued to show elevated Depression/Anxiety over the GE group. These results indicate that childhood carriers and noncarriers of the *mHTT* gene‐mutation are responding differently to similar environmental contexts. Increased Depression/Anxiety in GNE is arguably not surprising given the psychosocial burden of growing up in an HD family (Lewit‐Mendes et al., 2018; van der Meer et al., 2012). Children from HD families are at higher risk than the general population to be exposed to adverse childhood experiences, which in turn is associated with increased risk for anxiety and depression (Hanson et al., 2018; Telzer, 2016). The absence of depression traits in the GE group suggests that *mHTT* gene‐mutation carriers may have increased resiliency against psychosocial stress during childhood and adolescence.

Further, emotional resiliency in GE children and adolescence may be related to differences in striatal development. The ventral striatum has been established as a key structure in emotional processing (Hanson et al., 2018; Telzer, 2016). Mouse models indicate that chronic psychosocial stress normally activates the striatum, and striatal activation is thought to function in distinguishing reward from punishment during motivational learning (Enzi et al., 2012; Laine et al., 2017). During childhood, differences in striatal activation in response to reward are predictive of depressive symptomology (Telzer et al., 2014). In pre‐HD adults, functional magnetic resonance imaging (fMRI) findings indicate a lack of distinct striatal activity when anticipating motivational and control stimuli (Enzi et al., 2012), possibly pointing to differences in motivational processing that precedes motor deficits and explain decreased susceptibility to anxiety and depression symptomology.

The notion that mHTT may benefit brain function in children and adolescent carriers is consistent with our previous work from the Kids‐HD study evaluating effects of HTT on brain development where we report increasing CAG repeats driving higher cognitive skill, a pattern seen throughout the spectrum of repeats and into the expanded range (Schultz, van der Plas, et al., 2021). We also recently reported the same findings in a sample from the large ENROLL data base where higher repeats were associated with higher cognitive skill in premanifest young adults (Schultz, Saft, et al., 2021). Similarly, *mHTT* carriers show evidence of striatal‐cerebellar hyperconnectivity during childhood and adolescence (A. V. Tereshchenko et al., 2020). These findings are consistent with the theory that the polymorphism of CAG repeats in the HTT gene was positively selected for human brain evolution and posits that the advantageous changes created early in life are then associated with the disadvantage of degeneration later in life (van der Plas et al., 2020). It may be that *mHTT* drives the development of a striatal circuit that provides superior function early in life, yet this circuit is not built to last and later degenerates. This makes sense as advantages to brain function are typically only necessary up to reproductive age (Albin, 1988).

It is also possible that these results may be indicative of some emotional flattening in GE children. While apathy is more commonly associated with the cognitive decline in late‐stage HD, it has been detected in adult premanifest populations (Andrews et al., 2020; Labuschagne et al., 2013; Martinez‐Horta et al., 2016; Thieben et al., 2002). However, given that neurodegeneration of emotional‐processing striatal networks has also been observed in premanifest adults (Langley et al., 2021; Novak et al., 2012), it is unclear whether altered neurodevelopmental would contribute to early manifestation of apathy. Unfortunately, the PBS does not include any items that specifically evaluate apathy or emotional intelligence, so the contribution of apathy to lowered depression/anxiety in child and adolescent carriers requires further investigation.

The present study is not without limitations. First, limitations of the sample size may have contributed to low power, which is particularly pertinent when considering the lack of a CAG dose effect. Second, the PBS utilizes a demographically homogeneous and dated normative sample (Lindgren & Koeppl, 1987), limiting our ability to clinically interpret anxiety/depression levels in each group. Additionally, proxy measures are excellent at identifying observation‐based data related to behaviors, but use of self‐report is recommended to fully evaluate internalizing behaviors (Upton et al., 2008). While neither of these limitations change the validity of the observed group difference, it would be useful to replicate this study using more widely established used behavioral assessments, such as the Child Behavior Checklist or the Behavior Assessment System for Children. Expanding the assessment approach may serve to further illuminate group differences in emotional processing, as self‐reported assessments have demonstrated increased validity in detecting internalized symptoms (van de Looij‐Jansen et al., 2011). Notably, parents were unaware of their child's genetic status, which likely helped curb response bias.

Our findings highlight the importance of considering both psychosocial stress and brain developmental trajectories when evaluating neuropsychiatric symptoms in children and adolescents. Based on elevated incidence of depression/anxiety among noncarrier children and adolescents from HD families, compared to normative samples, future studies should aim to clarify clinical burden, identify causal factors, and guide recommendations for additional psychosocial support. Additionally, age‐dependent changes in neuropsychiatric risk should be further elucidated through longitudinal measures. The differences between GNE and GE populations in both striatal development and anxiety/depression *t*‐scores suggest that susceptibility is modulated by gene status. While further research is warranted, we tentatively conclude that atypical striatal development in GE may reduce risk of depression/anxiety in childhood.

### PEER REVIEW

The peer review history for this article is available at https://publons.com/publon/10.1002/brb3.2630.

## Data Availability

De‐identified data can be shared upon reasonable request.
